# Phenotypic Heterogeneity in Crohn’s Disease-Associated Intestinal Strictures: An Exploratory Retrospective Cohort Study

**DOI:** 10.3390/diagnostics16121841

**Published:** 2026-06-14

**Authors:** Stefano Fusco, Juliette Nesseler, Lisa Minn, Sabrina Groß, Nisar P. Malek, Christoph R. Werner

**Affiliations:** 1 Department of Internal Medicine, Gastroenterology, Oncology and Palliative Care, Medical Campus Lake Constance, Röntgenstraße 2, 88048 Friedrichshafen, Germany; 2 Department of Gastroenterology, Gastrointestinal Oncology, Hepatology, Infectious Diseases and Geriatrics, University Hospital Tübingen, Otfried-Müller-Straße 10, 72076 Tübingen, Germanylisa.minn@med.uni-tuebingen.de (L.M.); sabrina.gross@med.uni-tuebingen.de (S.G.); nisar.malek@med.uni-tuebingen.de (N.P.M.); christoph.werner@med.uni-tuebingen.de (C.R.W.)

**Keywords:** Crohn’s disease, intestinal stricture, fibrosis, intestinal inflammation

## Abstract

**Background**: Crohn’s disease-associated intestinal strictures represent a major source of morbidity and frequently require endoscopic or surgical intervention. However, patients with stricturing Crohn’s disease demonstrate substantial clinical heterogeneity regarding disease localization, penetrating complications, systemic manifestations, metabolic alterations, and treatment exposure. This study aimed to explore phenotypic heterogeneity within patients with Crohn’s disease-associated intestinal strictures. **Methods**: In this retrospective exploratory cohort study, 96 patients with Crohn’s disease-associated intestinal strictures treated at a tertiary referral center between 2014 and 2024 were included. Clinical, structural, metabolic, and treatment-related variables were analyzed. Univariate analyses were performed using chi-square, Fisher’s exact test, Student’s *t*-test, or Mann–Whitney U test as appropriate. Exploratory multivariable logistic regression models were constructed to explore relationships between different clinical phenotypes and disease-related characteristics, including extraintestinal manifestations (EIMs), smoking status, penetrating disease manifestations, hepatic steatosis, stenosis localization, and abscess formation. Given the limited sample size and event numbers in several subgroup analyses, all multivariable analyses were considered exploratory and hypothesis-generating. **Results**: The cohort demonstrated a heterogeneous clinical presentation with a high prevalence of perianal disease, penetrating complications, prior intestinal surgery, and biologic therapy exposure. Female sex (OR 4.63, *p* = 0.044), autoimmune disease (OR 23.5, *p* = 0.049), rectal stenosis (inverse association; OR 0.08, *p* = 0.041), and exposure to multiple biologic therapies (OR 20.11, *p* = 0.036) remained associated with EIMs after multivariable adjustment. Smoking status was associated with anastomotic stenosis (OR 3.16, *p* = 0.023) and inversely associated with female sex (OR 0.35, *p* = 0.036). Phenotype-oriented analyses further suggested clustering of penetrating disease manifestations, including associations between intestinal fistulas, perianal fistulas, and abscess formation. Hepatic steatosis demonstrated exploratory associations with intestinal fistulas, intestinal resection, and appendectomy. Several analyses demonstrated wide confidence intervals and should therefore be interpreted cautiously. **Conclusions**: This exploratory retrospective cohort study highlights the substantial clinical heterogeneity observed among patients with Crohn’s disease-associated intestinal strictures. Different structural, systemic, penetrating, behavioral, and metabolic disease manifestations may indicate potentially overlapping phenotypic patterns within stricturing Crohn’s disease. Given the retrospective design, limited sample size, and exploratory statistical approach, these findings should be interpreted cautiously and require validation in larger prospective studies.

## 1. Introduction

Crohn’s disease (CD) is a chronic, immune-mediated inflammatory bowel disease characterized by transmural inflammation and progressive structural damage of the intestinal wall. Despite major advances in medical therapy over recent decades, intestinal strictures continue to represent one of the most frequent and clinically relevant complications of CD. They are a major cause of bowel obstruction, impaired quality of life, and repeated need for endoscopic or surgical intervention [1,2]. Epidemiological data consistently demonstrate that fibrostenotic complications account for a substantial proportion of long-term morbidity in patients with CD [3].

The clinical course of Crohn’s disease typically evolves over time. While many patients initially present with an inflammatory disease phenotype, the risk of developing stricturing or penetrating complications increases with disease duration. Longitudinal cohort studies have shown that 30–50% of CD patients develop intestinal strictures within the first decade after diagnosis, often necessitating mechanical intervention [3,4]. Several factors have been associated with an increased risk of stricture formation, including ileal disease location, early age at disease onset, cigarette smoking, prolonged uncontrolled inflammation, and delayed initiation of effective therapy [3,4,5]. In addition, disease-related characteristics such as deep transmural inflammation and recurrent disease flares appear to accelerate fibrotic remodeling of the bowel wall. Together, these observations are consistent with the concept that fibrostenotic Crohn’s disease may reflect cumulative bowel damage over time rather than a distinct disease subtype, underscoring the importance of early risk stratification and timely therapeutic intervention.

From a pathophysiological perspective, Crohn’s disease–associated strictures arise from a complex interaction between chronic inflammation, dysregulated wound-healing responses, progressive intestinal fibrosis and gut microbiota [6]. Persistent inflammatory activity promotes activation of fibroblasts and myofibroblasts, leading to excessive extracellular matrix deposition, smooth muscle hyperplasia, and irreversible architectural remodeling of the bowel wall [7,8,9,10]. Central profibrotic pathways, including transforming growth factor-β-mediated signaling and alterations in matrix turnover, play a key role in this process [7,8]. Importantly, experimental and clinical evidence suggests that fibrogenesis may become partially self-sustaining and continue even after mucosal inflammation has been adequately controlled [10,11].

Clinically and histopathologically, intestinal strictures in CD are heterogeneous. Most lesions display mixed inflammatory and fibrotic components, whereas purely inflammatory or purely fibrotic strictures are relatively uncommon [1,10]. This heterogeneity has direct implications for clinical management. While strictures with a substantial inflammatory component may respond to escalation of anti-inflammatory or biologic therapy, predominantly fibrotic strictures are typically refractory to medical treatment and often require endoscopic or surgical intervention [11,12]. Accurate phenotyping of strictures therefore remains a central challenge in the management of fibrostenosing Crohn’s disease.

Endoscopy plays a key role in the assessment of luminal narrowing and mucosal disease activity but is limited in its ability to evaluate transmural and extramural pathology. As a result, cross-sectional imaging has become integral to the diagnostic workup of patients with suspected or established stricturing disease. Magnetic resonance enterography (MRE), computed tomography enterography (CTE), and intestinal ultrasound allow assessment of stricture location, length, bowel wall thickness, prestenotic dilatation, and surrogate markers of inflammation [13,14]. Among these modalities, MRE and intestinal ultrasound are increasingly preferred due to their high diagnostic accuracy and lack of ionizing radiation [15,16]. Nevertheless, conventional imaging parameters remain insufficient for reliably distinguishing inflammatory from fibrotic tissue, particularly in strictures with mixed features [17].

Current therapeutic strategies for Crohn’s disease-associated strictures include optimization of anti-inflammatory medical therapy, endoscopic interventions, and surgery [18]. Endoscopic balloon dilation offers a bowel-sparing option for selected short strictures and has demonstrated acceptable short-term outcomes, although recurrence and the need for repeat procedures are common [12,19,20]. Surgical management, including resection or strictureplasty, remains definitive for many patients with fibrostenotic disease but is associated with postoperative recurrence and cumulative bowel loss [21,22]. Notably, although the efficacy and safety of anti-TL1A antibodies are currently being evaluated in several ongoing studies, there is still no approved anti-fibrotic therapy specifically targeting intestinal fibrosis, highlighting a major unmet need in the management of Crohn’s disease [23,24].

In addition to structural and penetrating complications, Crohn’s disease has increasingly been associated with systemic metabolic and hepatobiliary manifestations, including hepatic steatosis and biliary disease. Chronic systemic inflammation, cumulative disease burden, intestinal surgery, nutritional alterations, and long-term medical therapy may contribute to these manifestations. Previous studies have demonstrated an increased prevalence of non-alcoholic fatty liver disease (NAFLD) in patients with inflammatory bowel disease, particularly Crohn’s disease, although data specifically addressing stricturing Crohn’s disease phenotypes remain limited [25,26,27].

Although stricturing Crohn’s disease is commonly discussed as a single disease phenotype, patients with intestinal strictures frequently demonstrate substantial clinical heterogeneity regarding disease localization, penetrating complications, systemic manifestations, metabolic alterations, and treatment exposure. However, comprehensive phenotypic characterization within stricturing Crohn’s disease remains insufficiently investigated in real-world cohorts.

Several clinical factors have previously been associated with stricturing Crohn’s disease phenotypes, including ileal disease location, smoking, penetrating disease behavior, prior intestinal surgery, and chronic inflammatory activity [28,29]. In addition, progressive intestinal fibrosis and fibrostenotic remodeling are increasingly recognized as dynamic and heterogeneous processes within Crohn’s disease progression [30]. However, the interaction between systemic, structural, penetrating, behavioral, and metabolic disease domains within patients with established stricturing Crohn’s disease remains insufficiently characterized.

Accordingly, the present study was designed to explore how these systemic, structural, penetrating, behavioral, and metabolic disease domains interact within patients with established stricturing Crohn’s disease.

Therefore, the primary aim of this exploratory retrospective cohort study was not to identify predictors of stricture development, but rather to explore phenotypic heterogeneity within patients with established Crohn’s disease-associated intestinal strictures by analyzing systemic, structural, penetrating, behavioral, and metabolic disease manifestations.

## 2. Materials and Methods

### 2.1. Study Design and Patient Population

**Figure 1:**
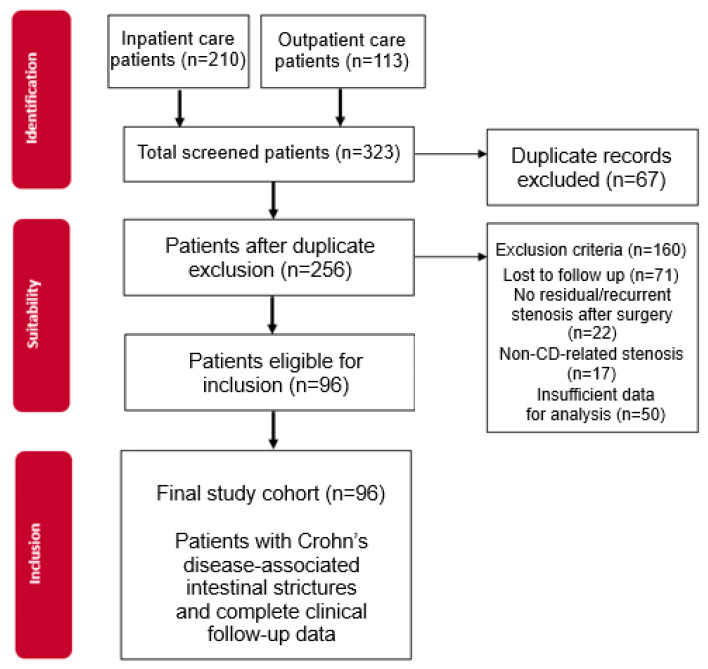
Flow chart illustrating patient identification, exclusion criteria, and final cohort selection.

This retrospective cohort study included patients with Crohn’s disease-associated intestinal strictures who were treated at a tertiary referral center between 2014 and 2024. Patients were identified through retrospective screening of institutional electronic medical records, endoscopy reports, radiological databases, and discharge documentation at the University Hospital Tübingen between 2014 and 2024. Initially, all patients with a documented diagnosis of Crohn’s disease and suspected intestinal strictures were screened. Intestinal strictures were defined as persistent luminal narrowing with associated prestenotic dilatation, obstructive symptoms, and/or impaired endoscopic passage documented on endoscopy or cross-sectional imaging, including magnetic resonance enterography (MRE), computed tomography (CT), or intestinal ultrasound. In cases where both endoscopic and radiological data were available, classification was based on the overall clinical assessment documented in the medical records. Patients were excluded if no confirmed intestinal stricture was documented on endoscopy or cross-sectional imaging, if the diagnosis of Crohn’s disease could not be confirmed based on established clinical, radiological, endoscopic, and histopathological criteria, or if clinical data were incomplete. After application of the inclusion and exclusion criteria, 96 patients with confirmed Crohn’s disease-associated intestinal strictures were included in the final analysis. A flow chart illustrating patient selection is provided in Figure 1. Patients without evidence of intestinal strictures or with insufficient clinical data were excluded.

### 2.2. Data Collection

Clinical, demographic, and treatment-related data were extracted from electronic medical records. The following variables were recorded:

- Demographic data: age, sex, body mass index (BMI);
- Disease characteristics: disease duration, disease location, presence of rectal and ileal stenosis;
- Complications: perianal disease, perianal fistulas, intestinal fistulas, abscess formation;
- Extraintestinal manifestations (EIMs) and autoimmune diseases;
- Surgical history: intestinal resection, appendectomy;
- Imaging findings: hepatic steatosis, cholecystolithiasis;
- Treatment variables: biologic therapy, number of biologics, specific agents (e.g., infliximab);
- Lifestyle factors: smoking status.

Extraintestinal manifestations (EIMs) included musculoskeletal, dermatologic, ophthalmologic, and hepatobiliary manifestations documented in the electronic medical records according to established clinical criteria. Autoimmune diseases were defined based on documented physician diagnoses recorded in the institutional medical records and included autoimmune hepatobiliary diseases such as autoimmune hepatitis (AIH), primary biliary cholangitis (PBC), primary sclerosing cholangitis (PSC), and autoimmune pancreatitis (AIP), as well as autoimmune thyroiditis and selected rheumatologic/systemic autoimmune disorders, including rheumatoid arthritis, ankylosing spondylitis, psoriatic arthritis, systemic lupus erythematosus, and Sjögren syndrome. Hepatic steatosis and cholecystolithiasis were assessed based on imaging findings (ultrasound, CT, or MRI). A multivariate model was constructed to assess factors associated with perianal fistulizing disease. Variables included other manifestations of penetrating disease, including intestinal fistulas and abscess formation. Intestinal ulceration was defined as endoscopically documented mucosal ulceration in the affected bowel segment.

### 2.3. Outcomes

The selected exploratory outcomes were chosen to represent clinically relevant systemic, structural, penetrating, behavioral, and metabolic disease domains within stricturing Crohn’s disease. The primary objective of this study was not to identify predictors of stricture development, but rather to explore associations between different phenotypic manifestations within stricturing Crohn’s disease. These domains were selected to capture clinically relevant dimensions of disease heterogeneity frequently encountered in stricturing Crohn’s disease.

The following outcomes were analyzed using separate models:

- Extraintestinal manifestations (EIMs);
- Smoking status;
- Perianal fistulas;
- Hepatic steatosis;
- Stenosis location (rectal and ileal stenosis);
- Abscess formation.

### 2.4. Statistical Analysis

Continuous variables are presented as mean ± standard deviation (SD) or median with interquartile range (IQR), depending on data distribution. Categorical variables are expressed as absolute numbers and percentages.

For univariate analyses, comparisons between groups were performed using the chi-square test or Fisher’s exact test for categorical variables, and Student’s *t*-test or Mann–Whitney U test for continuous variables, as appropriate.

The multivariable analyses were designed as exploratory phenotype-oriented models rather than predictive risk models. Separate regression models were constructed to evaluate clinically relevant systemic, structural, penetrating, behavioral, and metabolic disease domains within stricturing Crohn’s disease. Variables with a *p*-value < 0.10 in univariate analysis and variables considered clinically relevant were included in the multivariable models. Variable selection was based on clinical relevance, biological plausibility, and statistical significance in univariate analyses. Results of logistic regression analyses are reported as odds ratios (OR) with 95% confidence intervals (CI) and corresponding *p*-values. To reduce excessive model complexity, the number of variables included in each model was restricted relative to the number of outcome events. Missing data were handled by case-wise exclusion, resulting in minor variations in sample size across analyses.

Given the limited sample size and event numbers in several subgroup analyses, the multivariable analyses should be interpreted cautiously. The models were exploratory and hypothesis-generating, and formal model stability analyses were not performed.

### 2.5. Ethical Considerations

The study was conducted in accordance with the Declaration of Helsinki and was approved by the local ethics committee (245/2023BO2). Due to the retrospective design, the requirement for informed consent was waived.

## 3. Results

### 3.1. Patient Characteristics and Univariate Analysis According to EIM and Smoking Status

A total of 96 patients with Crohn’s disease-associated intestinal strictures were included. Baseline characteristics and univariate comparisons according to extraintestinal manifestations (EIMs) and smoking status are presented in Table 1.

The cohort demonstrated a heterogeneous clinical profile, with a mean age of 60.6 ± 13.1 years and a female proportion of 45.8%. A high prevalence of complicated disease behavior was observed, including perianal disease (48.3%), fistulizing complications, and prior intestinal surgery. Patients with EIMs differed significantly from those without EIMs in several aspects. Female sex was more frequent among patients with EIMs (58.5% vs. 36.4%, *p* = 0.039). In addition, perianal disease was significantly more prevalent in patients with EIMs (59.2% vs. 34.2%, *p* = 0.030). Rectal stenosis was significantly less frequent in patients with EIMs (2.4% vs. 23.6%, *p* = 0.003). Autoimmune diseases were markedly more frequent in patients with EIMs (22.0% vs. 3.6%, *p* = 0.008).

Therapy-related variables also differed significantly. Patients with EIMs were more likely to receive biologic therapy (65.9% vs. 43.6%, *p* = 0.039) and to have been exposed to multiple biologics (46.3% vs. 23.6%, *p* = 0.028). Regarding smoking status, significant differences were observed for sex distribution, with a higher proportion of males among smokers (*p* = 0.039). Anastomotic stenosis was significantly more frequent in smokers (70.8% vs. 43.8%, *p* = 0.020).

### 3.2. Univariate Analysis According to Hepatic Steatosis and Fistulizing Disease

As shown in Table 2, patients with hepatic steatosis demonstrated a trend toward higher age (63.3 ± 12.4 vs. 58.9 ± 13.3 years, *p* = 0.089). Significant associations were observed for intestinal fistulas (48.6% vs. 19.7%, *p* = 0.005) and perianal fistulas (74.3% vs. 42.9%, *p* = 0.025). In addition, ileal stenosis was more common in patients with steatosis (82.9% vs. 62.3%, *p* = 0.040). Cholecystolithiasis was strongly associated with hepatic steatosis (25.7% vs. 3.3%, *p* = 0.002). Fistulizing disease was associated with perianal disease, abscess formation, intestinal fistulas, and longer disease duration in univariate analyses. Perianal disease was markedly more frequent in patients with fistulas (72.7% vs. 23.3%, *p* < 0.001). Similarly, abscess formation was significantly associated with fistulas (81.0% vs. 31.4%, *p* < 0.001), as were intestinal fistulas (45.8% vs. 14.6%, *p* = 0.002). Furthermore, longer disease duration (>10 years) was significantly associated with fistula presence (70.8% vs. 43.8%, *p* = 0.013).

### 3.3. Univariate Analysis According to Stenosis Location

Table 3 summarizes univariate comparisons according to rectal and ileal stenosis. Rectal stenosis was associated with higher frequencies of perianal disease, perianal fistulas, and abscess formation. Patients with rectal stenosis more frequently had perianal disease (84.6% vs. 41.9%, *p* = 0.006), perianal fistulas (64.3% vs. 31.7%, *p* = 0.033), and abscess formation (78.6% vs. 41.5%, *p* = 0.018). In addition, EIMs were significantly less frequent in patients with rectal stenosis (7.1% vs. 48.8%, *p* = 0.003). Ileal stenosis was associated with female sex (53.7% vs. 27.6%, *p* = 0.025) and biologic therapy (61.2% vs. 34.5%, *p* = 0.025). Hepatic steatosis was also more frequent in patients with ileal stenosis (43.9% vs. 20.7%, *p* = 0.040).

### 3.4. Univariate Analysis According to Abscess Formation

Univariate comparisons according to abscess formation are presented in Table 4. Abscesses were strongly associated with perianal disease (78.6% vs. 15.6%, *p* < 0.001), fistulizing disease (97.1% vs. 29.6%, *p* < 0.001), and intestinal fistulas (46.7% vs. 15.6%, *p* = 0.007). Perianal fistulas were also significantly more frequent in patients with abscesses (62.2% vs. 15.6%, *p* < 0.001). Rectal stenosis was significantly associated with abscess formation (24.4% vs. 3.1%, *p* = 0.012). Infliximab exposure was more frequent among patients with abscess formation (33.3% vs. 9.4%, *p* = 0.016).

### 3.5. Exploratory Multivariate Analysis of Systemic Disease Manifestations

To further characterize systemic disease manifestations within stricturing Crohn’s disease, exploratory multivariable analysis of extraintestinal manifestations (EIMs) was performed. Exploratory multivariable regression analysis was performed to evaluate associations between systemic disease manifestations and clinical disease characteristics within stricturing Crohn’s disease (Table 5). The model included clinically relevant variables identified in univariate analyses and suggested several associations within the exploratory model. Female sex remained associated within the exploratory model with EIMs after multivariable adjustment (OR 4.63, 95% CI 1.04–20.63, *p* = 0.044). Autoimmune disease remained associated with EIMs after multivariable adjustment, although the wide confidence intervals likely reflect the limited event numbers (OR 23.5, 95% CI 1.02–567.6, *p* = 0.049). In contrast, rectal stenosis remained inversely associated with EIMs after adjustment, with lower odds of EIMs (OR 0.08, 95% CI 0.01–0.90, *p* = 0.041). Furthermore, exposure to multiple biologic therapies was associated with EIMs (OR 20.11, 95% CI 1.21–334.3, *p* = 0.036). Perianal disease did not retain statistical significance in the multivariate model. Given the wide confidence intervals and limited event numbers, these exploratory associations should be interpreted cautiously.

### 3.6. Exploratory Multivariate Analysis of Smoking-Associated Phenotypes

Multivariate analysis for smoking status identified both demographic and structural variables associated with smoking behavior (Table 6). Female sex remained associated with lower odds of smoking (OR 0.35, 95% CI 0.13–0.93, *p* = 0.036). In contrast, anastomotic stenosis was associated with smoking (OR 3.16, 95% CI 1.17–8.53, *p* = 0.023). Cholecystolithiasis showed a trend toward association with smoking (OR 3.22, 95% CI 0.89–11.67, *p* = 0.076), although this did not reach statistical significance.

### 3.7. Exploratory Multivariate Analysis of Penetrating Disease Manifestations

To further characterize penetrating disease behavior within the cohort, exploratory analyses of intestinal and perianal fistulas were conducted. Exploratory multivariable regression analysis was performed to evaluate clustering patterns among penetrating disease manifestations within stricturing Crohn’s disease (Table 7). Intestinal fistulas remained associated with perianal fistulas within the exploratory model after multivariable adjustment (OR 11.82, 95% CI 2.96–47.21, *p* < 0.001). Likewise, abscess formation remained associated with perianal fistulas within the exploratory model (OR 19.54, 95% CI 4.63–82.51, *p* < 0.001). In contrast, disease duration >10 years was inversely associated with perianal fistulas (OR 0.24, 95% CI 0.07–0.78, *p* = 0.018). Other variables included in the model did not retain statistical significance after adjustment. Despite statistically significant associations, the exploratory nature of the analyses and limited event numbers warrant cautious interpretation.

### 3.8. Exploratory Multivariate Analysis of Metabolic and Hepatobiliary Manifestations

To evaluate metabolic and hepatobiliary disease domains within stricturing Crohn’s disease, exploratory multivariable analysis of hepatic steatosis was performed. Exploratory multivariable regression analysis was performed to evaluate associations between metabolic and hepatobiliary manifestations and clinical disease characteristics within stricturing Crohn’s disease (Table 8). Intestinal fistulas remained associated with hepatic steatosis after multivariable adjustment (OR 28.91, 95% CI 1.94–431.4, *p* = 0.015). Prior intestinal resection was also associated with hepatic steatosis (OR 26.88, 95% CI 1.02–709.0, *p* = 0.049), as was appendectomy (OR 17.43, 95% CI 1.31–231.8, *p* = 0.030). Other variables included in the model did not remain statistically significant after adjustment. Given the wide confidence intervals and limited event numbers, these findings should be interpreted cautiously within the exploratory framework of the present analyses.

### 3.9. Exploratory Multivariate Analysis of Stenosis Localization Patterns

Multivariate logistic regression analyses were performed to identify independent factors associated with rectal and ileal stenosis (Table 9). For rectal stenosis, ileal stenosis was associated with a significantly lower likelihood of rectal involvement (OR 0.18, 95% CI 0.04–0.82, *p* = 0.027). Extraintestinal manifestations (EIMs) were not significantly associated with rectal stenosis (OR 0.18, 95% CI 0.02–1.67, *p* = 0.180).

A positive, but not statistically significant association was observed for abscess formation (OR 7.56, 95% CI 0.73–78.80, *p* = 0.091). Perianal fistulas did not seem to be associated with rectal stenosis (OR 1.77, 95% CI 0.33–9.55, *p* = 0.506).

For ileal stenosis, female sex was associated with increased odds, reaching borderline statistical significance (OR 2.84, 95% CI 1.02–8.17, *p* = 0.050). In contrast, rectal stenosis remained inversely associated with ileal stenosis after adjustment (OR 0.17, 95% CI 0.04–0.64, *p* = 0.009). Biologic therapy showed a positive but non-significant association with ileal stenosis (OR 2.63, 95% CI 0.95–7.28, *p* = 0.063). Similarly, hepatic steatosis demonstrated a positive trend (OR 2.61, 95% CI 0.87–7.77, *p* = 0.086), although neither reached statistical significance after adjustment. The observed associations should be interpreted cautiously due to limited subgroup sizes and statistical uncertainty.

### 3.10. Exploratory Multivariate Analysis of Abscess-Associated Disease Manifestations

Multivariate logistic regression analysis identified several independent factors associated with abscess formation (Table 10). Perianal disease remained associated with abscess formation, demonstrating a markedly increased odds (OR 30.18, 95% CI 2.55–356.7, *p* = 0.007). Similarly, the presence of any fistula was associated with abscess formation (OR 27.30, 95% CI 1.40–533.3, *p* = 0.029). Body mass index (BMI > 25 kg/m^2^) was also associated with abscess formation (OR 23.69, 95% CI 1.23–454.9, *p* = 0.036). In contrast, infliximab therapy was not associated with abscess formation (OR 1.11, 95% CI 0.12–10.57, *p* = 0.931). Perianal disease, any fistula, and BMI > 25 kg/m^2^ remained associated with abscess formation after multivariable adjustment. The wide confidence intervals may reflect the limited number of events and should be interpreted with caution.

## 4. Discussion

In this retrospective cohort study of patients with Crohn’s disease-associated intestinal strictures, we demonstrate that stricturing disease represents a highly heterogeneous and multifactorial clinical phenotype. Our findings highlight that systemic immune activity, structural complications, behavioral factors, and metabolic alterations interact in a complex manner and contribute to disease expression in distinct but overlapping ways. Intestinal strictures are a major complication of Crohn’s disease, occurring in up to one-third of patients during the disease course and frequently leading to repeated interventions and surgery [31,32]. The pathogenesis of stricture formation is driven by chronic and recurrent inflammation, which induces progressive tissue remodeling characterized by fibroblast activation, extracellular matrix deposition, and smooth muscle cell hyperplasia [32,33]. Importantly, accumulating evidence suggests that fibrotic processes may become partially independent of active inflammation, which explains why anti-inflammatory therapies are often insufficient to prevent or reverse established strictures [32,34]. This concept is particularly relevant when interpreting our findings, as several associations may reflect chronic disease burden rather than acute inflammatory activity.

A central observation of our study is the marked heterogeneity within the stricturing Crohn’s disease population. This aligns with current concepts that Crohn’s disease should be understood as a spectrum of disease phenotypes rather than a single entity [35]. Strictures frequently contain a mixture of inflammatory, fibrotic, and muscular components, as well as the gut microbiota, and their clinical presentation depends on the relative contribution of these processes, which can be detected using MRI or CT enterography [23,36,37].

The inverse association between rectal and ileal stenosis observed in our analysis suggests that disease localization may reflect distinct pathogenic pathways. Previous studies have demonstrated that disease behavior evolves over time and is influenced by genetic susceptibility, environmental factors, and immune responses [35,38]. Our findings are consistent with the hypothesis that stricturing disease is not uniform but consists of location-specific phenotypes with potentially different underlying mechanisms.

Extraintestinal manifestations (EIMs) represent an important component of Crohn’s disease and are reported in up to 40% of patients [39,40]. They are thought to arise from shared immunological pathways between intestinal and extraintestinal tissues, including dysregulated T-cell responses and cytokine signaling [41]. In our cohort, EIMs were associated with autoimmune comorbidities and exposure to multiple biologic therapies, suggesting that they may reflect a subgroup of patients with more severe and systemic disease. This is consistent with previous studies demonstrating that patients with EIMs have a more complicated disease course and are more likely to require treatment escalation [39]. The association between EIMs and systemic immune activation is consistent with the concept of Crohn’s disease as a systemic inflammatory disorder rather than a purely intestinal disease. Interestingly, we observed no strong association between EIMs and certain localized structural features, suggesting that systemic immune activity and local fibrostenotic processes may be partially dissociated.

Smoking is one of the most consistently identified environmental risk factors influencing Crohn’s disease progression. It has been associated with increased disease activity, higher rates of relapse, and a greater need for surgery [4,41]. In our study, smoking was independently associated with anastomotic stenosis, indicating a potential role in postoperative disease recurrence and impaired healing. Mechanistically, smoking affects mucosal immunity, alters cytokine profiles, and influences the intestinal microbiome [41]. These effects may promote both inflammation and fibrosis, thereby contributing to structural complications. The inverse association with female sex observed in our cohort may reflect epidemiological differences in smoking behavior rather than biological effects.

The strong association between fistulas and abscess formation observed in our study underscores the close relationship between penetrating disease behavior and septic complications. These findings are consistent with the concept that stricturing and penetrating disease represent overlapping rather than distinct phenotypes [35,42]. Transmural inflammation plays a central role in the development of fistulas and abscesses, leading to tissue destruction and abnormal connections between intestinal segments or adjacent structures [42]. The observed association between infliximab exposure and abscess formation should be interpreted cautiously, as treatment exposure was assessed retrospectively and may reflect confounding by indication rather than a causal relationship. Infliximab therapy may have been initiated after adequate infection control or in patients with previously treated fistulizing disease. The coexistence of fistulizing and stricturing features in our cohort is consistent with the concept of advanced disease characterized by cumulative structural damage. These findings highlight that different manifestations of penetrating Crohn’s disease frequently co-occur, supporting the concept of a shared underlying pathophysiological mechanism. An important and increasingly recognized aspect of Crohn’s disease is the interaction between metabolic factors and inflammatory pathways. In our study, hepatic steatosis was associated with markers of disease severity, including prior surgery. Hepatic steatosis has been reported in up to 40% of patients with inflammatory bowel disease and may be related to chronic inflammation, altered lipid metabolism, and changes in the gut–liver axis [25,43]. Mesenteric adipose tissue, often referred to as “creeping fat,” plays an active role in Crohn’s disease by secreting pro-inflammatory cytokines and adipokines, thereby contributing to both inflammation and fibrosis [44]. Our findings are consistent with the hypothesis that metabolic alterations are not merely comorbidities but may be integrated into the pathophysiology of fibrostenotic disease.

The exploratory associations observed between hepatic steatosis and selected disease characteristics may reflect the complex interplay between chronic systemic inflammation, cumulative disease burden, intestinal surgery, nutritional alterations, and metabolic dysregulation in Crohn’s disease. Previous studies have demonstrated an increased prevalence of NAFLD and hepatobiliary manifestations in patients with inflammatory bowel disease, particularly Crohn’s disease [25,26,27]. Moreover, disease severity, bowel surgery, and inflammatory burden have previously been associated with hepatic steatosis in IBD cohorts [45,46]. However, data specifically addressing metabolic and hepatobiliary alterations in stricturing Crohn’s disease phenotypes remain limited. Therefore, these findings should primarily be interpreted as exploratory and hypothesis-generating.

The accurate characterization of intestinal strictures remains a major challenge in clinical practice. Endoscopy provides limited information on transmural disease, whereas cross-sectional imaging modalities such as magnetic resonance enterography (MRE) and computed tomography enterography (CTE) allow assessment of bowel wall thickness, enhancement patterns, and extramural complications [13,36].

However, distinguishing between inflammatory and fibrotic components remains difficult, particularly in mixed lesions [36,47]. This limitation has important therapeutic implications, as treatment strategies differ significantly between inflammatory and fibrotic strictures. Emerging imaging techniques and artificial intelligence-based approaches may improve diagnostic accuracy in the future, but further validation is needed. The management of stricturing Crohn’s disease remains challenging and often requires a multidisciplinary approach [48]. While biologic therapies are effective in controlling inflammation, their impact on established fibrosis is limited [49,50]. This may be reflected in the high rates of progression to endoscopic or surgical intervention in patients with strictures. Endoscopic balloon dilation represents an effective bowel-sparing option for selected patients with short and accessible strictures, although recurrence rates remain substantial [35,51]. Surgical resection continues to play a central role in the management of fibrostenotic disease, but is associated with postoperative recurrence and cumulative bowel damage [52]. Recent advances in understanding the molecular mechanisms of fibrosis have identified potential targets for antifibrotic therapies, including pathways involved in fibroblast activation and extracellular matrix remodeling [23,53]. These approaches may represent a promising future direction for the treatment of stricturing Crohn’s disease.

Recent studies increasingly emphasize that fibrostenotic Crohn’s disease represents a heterogeneous and dynamic disease spectrum characterized by varying contributions of chronic inflammation, intestinal fibrosis, penetrating complications, systemic inflammatory manifestations, and metabolic comorbidities. Emerging concepts of phenotype-oriented disease stratification suggest that stricturing Crohn’s disease should no longer be considered a uniform fibrostenotic entity, but rather a multidimensional phenotype with substantial interindividual variability regarding disease behavior, progression, and therapeutic response [48,54]. In this context, the exploratory associations observed in the present study may support the concept of partially overlapping systemic, structural, penetrating, behavioral, and metabolic disease domains within stricturing Crohn’s disease. Recent advances in cross-sectional imaging, fibrosis assessment, and precision medicine approaches further highlight the importance of integrated phenotypic characterization to improve individualized therapeutic decision-making and risk stratification in Crohn’s disease [55]. However, despite increasing recognition of fibrostenotic heterogeneity, clinically validated biomarkers and standardized fibrosis-oriented phenotyping strategies remain limited. Therefore, prospective multicenter studies integrating clinical, imaging, molecular, and translational fibrosis markers are required to better define clinically meaningful fibrostenotic phenotypes and to support future phenotype-oriented management strategies in Crohn’s disease.

Taken together, these findings emphasize that stricturing Crohn’s disease should not be regarded as a uniform disease entity but rather as a multidimensional phenotype requiring individualized diagnostic and therapeutic strategies.

Taken together, the exploratory analyses performed in this study suggest that stricturing Crohn’s disease represents a multidimensional and clinically heterogeneous disease phenotype characterized by overlapping systemic, penetrating, behavioral, structural, and metabolic manifestations. Rather than representing isolated disease domains, these phenotypic patterns may interact within complex clinical trajectories of stricturing Crohn’s disease.

This study has several limitations. First, this was a retrospective single-center cohort study with a relatively limited sample size, which restricts the generalizability of the findings and increases the risk of selection bias. Second, the absence of a non-stricturing Crohn’s disease control cohort limits the ability to investigate predictors specifically associated with stricture development. Third, several exploratory multivariable analyses were performed despite limited event numbers in some subgroup analyses, increasing the possibility of overfitting and model instability. The wide confidence intervals observed in several regression models may further reflect the exploratory nature of the analyses and require cautious interpretation. In addition, missing data resulted in minor variations in sample size across analyses. Finally, the observational design does not permit causal inference. Future prospective multicenter studies with larger cohorts are required to validate these exploratory findings and further investigate phenotype heterogeneity in stricturing Crohn’s disease.

## 5. Conclusions

In conclusion, stricturing Crohn’s disease represents a heterogeneous and multifactorial phenotype characterized by complex interactions between systemic immune activity, structural complications, behavioral factors, and metabolic alterations. Our findings are consistent with a phenotype-driven approach to disease characterization and highlight the need for improved diagnostic tools and targeted antifibrotic therapies to optimize patient management.

## Figures and Tables

**Table 1:** Baseline characteristics and univariate comparisons according to extraintestinal manifestations and smoking status.

Variable	Total (*n* = 96)	EIMs	No EIMs	*p* Value	Total (*n* = 80)	Smoker	Non-Smoker	*p* Value
Age	60.6 (±13.1)	60.0 (±8.9)	61.0 (±15.6)	0.718	60.5 (±12.9)	61.3 (±12.7)	59.4 (13.5)	0.266
Female sex	44 (45.8)	24 (58.5)	20 (36.4)	0.039	40 (50.0)	19 (39.6)	21 (65.6)	0.039
BMI (>25 kg/m^2^)	33 (34.4)	12 (29.3)	21 (38.2)	0.393	69 (86.3)	40 (83.3)	29 (90.6)	0.511
smoking	48 (60.0)	23 (62.2)	25 (58.1)	0.820	–	–	–	–
EIMs	–	–	–	–	37 (46.3)	23 (47.9)	14 (43.8)	0.820
Perianal disease	42 (48.3)	13 (34.2)	29 (59.2)	0.030	34 (46.6)	22 (50.0)	12 (41.4)	0.485
Any fistula	48 (70.6)	20 (64.5)	28 (75.7)	0.424	40 (69.0)	24 (68.6)	16 (69.6)	1.000
Intestinal fistula	29 (51.8)	15 (65.2)	14 (42.4)	0.111	24 (51.1)	15 (51.7)	9 (50.0)	1.000
Perianal fistula	35 (62.5)	11 (47.8)	24 (72.7)	0.092	29 (61.7)	19 (65.5)	10 (55.6)	0.548
Abscess	45 (58.4)	17 (51.5)	28 (63.6)	0.352	39 (48.8)	22 (45.8)	17 (53.1)	0.649
Rectal stenosis Ileal stenosis	14 (14.6)	1 (2.4)	13 (23.6)	0.003	7 (8.8)	4 (8.3)	3 (9.4)	1.000
	67 (69.8)	30 (73.2)	37 (67.3)	0.654	58 (72.5)	34 (70.8)	24 (75.0)	0.800
Anastomosis stenosis	55 (57.3)	23 (56.1)	32 (58.2)	1.000	48 (60.0)	34 (70.8)	14 (43.8)	0.020
Inflammatory stenosis	8 (8.3)	4 (9.8)	4 (7.3)	0.720	7 (8.8)	2 (4.2)	5 (15.6)	0.109
Stenosis > 10 years	55 (57.3)	22 (53.7)	33 (60.0)	0.677	44 (55.0)	27 (56.3)	17 (53.1)	0.822
Intestinal resection	13 (13.5)	5 (12.2)	8 (14.5)	1.000	10 (12.5)	6 (12.5)	4 (12.5)	1.000
Appendectomy	22 (22.9)	12 (29.3)	10 (18.2)	0.227	21 (26.3)	13 (27.1)	8 (25.0)	1.000
Autoimmune disease	11 (11.5)	9 (22.0)	2 (3.6)	0.008	10 (12.5)	6 (12.5)	4(12.5)	1.000
Biologic therapy	1 (53.1)	27 (65.9)	24 (43.6)	0.039	45 (56.3)	28 (58.3)	17 (53.1)	0.654
Multiple biologics	32 (33.3)	19 (46.3)	13 (23.6)	0.028	27 (33.8)	14 (29.2)	13 (40.6)	0.339
Ustekinumab	24 (25.0)	14 (34.1)	10 (18.2)	0.096	22 (27.5)	13 (27.1)	9 (28.1)	1.000
Infliximab	26 (27.4)	14 (35.0)	12 (21.8)	0.170	21 (26.6)	15 (31.9)	6 (18.8)	0.299
Adalimumab	32 (33.3)	18 (43.9)	14 (25.5)	0.080	27 (33.8)	16 (33.3)	11 (34.4)	1.000
Vedolizumab	20 (20.8)	9 (22.0)	11 (20.0)	1.000	18 (22.5)	10 (20.8)	8 (25.0)	0.786
Hepatic steatosis	35 (36.5)	16 (39.0)	19 (34.5)	0.674	31 (38.8)	22 (45.8)	9 (28.1)	0.160
Cholecystolithiasis	11 (11.5)	3 (7.3)	8 (14.5)	0.343	8 (10.0)	2 (4.2)	6 (18.8)	0.054

Abbreviations: BMI, body mass index; EIMs, extraintestinal manifestations; OR, odds ratio; CI, confidence interval. Percentages are calculated based on available cases for each variable.

**Table 2:** Baseline characteristics and univariate comparisons according to hepatic steatosis and fistulizing disease.

Variable	Total (*n* = 96)	Hepatic Steatosis	No Hepatic Steatosis	*p* Value	Total (*n* = 80)	Fistula	No Fistula	*p* Value
Age (mean ± SD)	60.6 (±13.1)	63.3 (±12.4)	58.9 (±13.3)	0.089	60.5 (±12.9)	61.3 (±12.7)	59.4 (±13.5)	0.266
Female sex	44 (45.8)	16 (45.7)	28 (45.9)	1.000	44 (45.8)	20 (41.7)	24 (50.0)	0.539
BMI (>25 kg/m^2^)	33 (34.4)	14 (40.0)	19 (31.1)	0.384	33 (34.4)	19 (39.6)	14 (29.2)	0.390
smoking	48 (60.0)	22 (71.0)	26 (53.1)	0.160	48 (60.0)	24 (60.0)	24 (60.0)	1.000
EIMs	41 (42.7)	16 (45.7)	25 (41.0)	0.674	41 (42.7)	20 (41.7)	21 (43.8)	1.000
Perianal disease	42 (48.3)	11 (34.4)	31 (56.4)	0.074	42 (48.3)	32 (72.7)	10 (23.3)	<0.001
Any fistula	48 (50.0)	17 (48.6)	31 (50.8)	1.000	–	–	–	–
Intestinal fistula	29 (30.2)	17 (48.6)	12 (19.7)	0.005	29 (30.2)	22 (45.8)	7 (14.6)	0.002
Perianal fistula	35 (62.5)	9 (42.9)	26 (74.3)	0.025	35 (36.5)	29 (60.4)	6 (12.5)	<0.001
Abscess	45 (58.4)	15 (50.0)	30 (63.8)	0.247	45 (58.4)	34 (81.0)	11 (31.4)	<0.001
Rectal stenosis Ileal stenosis	14 (14.6)	3 (8.6)	11 (18.0)	0.245	14 (14.6)	9 (18.8)	5 (10.4)	0.386
	67 (69.8)	29 (82.9)	38 (62.3)	0.040	67 (69.8)	32 (66.7)	35 (72.9)	0.657
Anastomosis stenosis	55 (57.3)	22 (62.9)	33 (54.1)	0.521	55 (57.3)	28 (58.3)	27 (56.3)	1.000
Inflammatory stenosis	8 (8.3)	2 (5.7)	6 (9.8)	0.706	8 (8.3)	4 (8.3)	4 (8.3)	1.000
Stenosis > 10 years	55 (57.3)	20 (57.1)	35 (57.4)	1.000	55 (57.3)	21 (43.8)	34 (70.8)	0.013
Intestinal resection	13 (13.5)	8 (22.9)	5 (8.2)	0.062	13 (13.5)	8 (16.7)	5 (10.4)	0.552
Appendectomy	22 (22.9)	12 (34.3)	10 (16.4)	0.076	22 (22.9)	13 (27.1)	9 (18.8)	0.467
Autoimmune disease	11 (11.5)	3 (8.6)	8 (13.1)	0.741	11 (11.5)	6 (12.5)	5 (10.4)	1.000
Biologic therapy	51 (53.1)	20 (57.1)	31 (50.8)	0.671	51 (53.1)	23 (47.9)	28 (58.3)	0.413
Multiple biologics	32 (33.3)	9 (25.7)	23 (37.7)	0.267	32 (33.3)	17 (35.4)	15 (31.3)	0.829
Ustekinumab	24 (25.0)	10 (28.6)	14 (23.0)	0.626	24 (25.0)	11 (22.9)	13 (27.1)	0.814
Infliximab	26 (27.4)	10 (29.4)	16 (26.2)	0.812	26 (27.4)	14 (29.2)	12 (25.5)	0.819
Adalimumab	32 (33.3)	11 (31.4)	21 (34.4)	0.825	32 (33.3)	18 (37.5)	14 (29.2)	0.516
Vedolizumab	20 (20.8)	7 (20.0)	13 (21.3)	1.000	20 (20.8)	10 (20.8)	10 (20.8)	1.000
Hepatic steatosis	–	–	–	–	35 (36.5)	17 (35.4)	18 (37.5)	1.000
Cholecystolithiasis	11 (11.5)	9 (25.7)	2 (3.3)	0.002	11 (11.5)	6 (12.5)	5 (10.4)	1.000

Abbreviations: BMI, body mass index; EIMs, extraintestinal manifestations; OR, odds ratio; CI, confidence interval. Percentages are calculated based on available cases for each variable.

**Table 3:** Baseline characteristics and univariate comparisons according to rectal stenosis and ileal stenosis.

Variable	Total (*n* = 96)	Rectal Stenosis	No Rectal Stenosis	*p* Value	Total (*n* = 80)	Ileal Stenosis	No Ileal Stenosis	*p* Value
Age	60.6 (±13.1)	61.7 (±13.6)	60.4 (±13.1)	0.368	60.6 (±13.1)	61.5 (±13.1)	58.6 (±13.3)	0.161
Female sex	44 (45.8)	6 (42.9)	38 (46.3)	1.000	44 (45.8)	36 (53.7)	8 (27.6)	0.025
BMI (>25 kg/m^2^)	33 (34.4)	3 (21.4)	30 (36.6)	0.367	33 (34.4)	24 (35.8)	9 (31.0)	0.815
smoking	48 (60.0)	4 (57.1)	44 (60.3)	1.000	48 (60.0)	34 (58.6)	14 (63.6)	0.800
EIMs	41 (42.7)	1 (7.1)	40 (48.8)	0.003	41 (42.7)	30 (44.8)	11 (37.9)	0.654
Perianal disease	42 (48.3)	11 (84.6)	31 (41.9)	0.006	42 (48.3)	28 (46.7)	14 (51.9)	0.817
Any fistula	48 (70.6)	9 (100)	39 (66.1)	0.050	48 (70.6)	32 (68.1)	16 (76.2)	0.575
Intestinal fistula	29 (30.2)	5 (35.7)	24 (29.3)	0.754	29 (30.2)	23 (34.3)	6 (20.7)	0.230
Perianal fistula	35 (36.5)	9 (64.3)	26 (31.7)	0.033	35 (36.5)	23 (34.3)	12 (41.4)	0.645
Abscess	45 (46.9)	11 (78.6)	34 (41.5)	0.018	45 (46.9)	32 (47.8)	13 (44.8)	0.827
Intestinal ulceration	72 (75.0)	14 (100)	58 (70.7)	0.018	72 (75.0)	50 (74.6)	22 (75.9)	1.000
Rectal stenosis Ileal stenosis	–	–	–	–	14 (14.6)	5 (7.5)	9 (31.0)	0.005
	67 (69.8)	5 (35.7)	62 (75.6)	0.005	–	–	–	–
Anastomosis stenosis	55 (57.3)	8 (57.1)	47 (57.3)	1.000	55 (57.3)	40 (59.7)	15 (51.7)	0.506
Inflammatory stenosis	8 (8.3)	2 (14.3)	6 (7.3)	0.330	8 (8.3)	5 (7.5)	3 (10.3)	0.694
Stenosis > 10 years	55 (57.3)	8 (57.1)	47 (57.3)	1.000	55 (57.3)	37 (55.2)	18 (62.1)	0.654
Intestinal resection	13 (13.5)	2 (14.3)	11 (13.4)	1.000	13 (13.5)	10 (14.9)	3 (10.3)	0.748
Appendectomy	22 (22.9)	1 (7.1)	21 (25.6)	0.178	22 (22.9)	19 (28.4)	3 (10.3)	0.066
Autoimmune disease	11 (11.5)	1 (7.1)	10 (12.2)	1.000	11 (11.5)	8 (11.9)	3 (10.3)	1.000
Biologic therapy	51 (53.1)	7 (50.0)	44 (53.7)	1.000	51 (53.1)	41 (61.2)	10 (34.5)	0.025
Multiple biologics	32 (33.3)	6 (42.9)	26 (31.7)	0.541	32 (33.3)	25 (37.3)	7 (24.1)	0.245
Ustekinumab	24 (25.0)	3 (21.4)	21 (25.6)	1.000	24 (25.0)	18 (26.9)	6 (20.7)	0.614
Infliximab	26 (27.4)	6 (42.9)	20 (24.7)	0.197	26 (27.4)	20 (30.3)	6 (20.7)	0.455
Adalimumab	32 (33.3)	4 (28.6)	28 (34.1)	0.768	32 (33.3)	25 (37.3)	7 (24.1)	0.245
Vedolizumab	20 (20.8)	3 (21.4)	17 (20.7)	1.000	20 (20.8)	16 (23.9)	4 (13.8)	0.412
Hepatic steatosis	35 (36.5)	3 (21.4)	32 (39.0)	0.245	35 (36.5)	29 (43.9)	6 (20.7)	0.040
Cholecystolithiasis	11 (11.5)	3 (21.4)	8 (9.8)	0.199	11 (11.5)	9 (13.4)	2 (6.9)	0.496

Abbreviations: BMI, body mass index; EIMs, extraintestinal manifestations; OR, odds ratio; CI, confidence interval. Percentages may vary slightly across analyses due to missing data and case-wise exclusion.

**Table 4:** Baseline characteristics and univariate comparisons according to abscess.

Variable	Total (*n* = 96)	Abscess	No Abscess	*p* Value
Age	60.6 (±13.1)	61.7 (±13.6)	60.4 (±13.1)	0.368
Female sex	35 (45.5)	22 (48.9)	13 (40.6)	0.496
BMI (>25 kg/m^2^)	26 (33.8)	19 (42.2)	7 (21.9)	0.087
Smoking	38 (57.6)	22 (56.4)	16 (59.3)	1.000
EIMs	33 (42.9)	17 (37.8)	16 (50.0)	0.352
Perianal disease	38 (51.4)	33 (78.6)	5 (15.6)	<0.001
Any fistula	42 (67.7)	34 (97.1)	8 (29.6)	<0.001
Intestinal fistula	26 (33.8)	21 (46.7)	5 (15.6)	0.007
Perianal fistula	33 (42.9)	28 (62.2)	5 (15.6)	<0.001
Intestinal ulceration	59 (76.6)	36 (80.0)	23 (71.9)	0.426
Rectal stenosis Ileal stenosis	12 (15.6)	11 (24.4)	1 (3.1)	0.012
	53 (68.8)	32 (71.1)	21 (65.6)	0.627
Anastomosis stenosis	45 (58.4)	30 (66.7)	15 (46.9)	0.103
Inflammatory stenosis	8 (10.4)	5 (11.1)	3 (9.4)	1.000
Stenosis > 10 years	47 (61.0)	24 (53.3)	23 (71.9)	0.154
Intestinal resection	10 (13.0)	7 (15.6)	3 (9.4)	0.509
Appendectomy	20 (26.0)	12 (26.7)	8 (25.0)	1.000
Autoimmune disease	10 (13.0)	5 (11.1)	5 (15.6)	0.733
Biologic therapy	41 (53.2)	25 (55.6)	16 (50.0)	0.651
Multiple biologics	25 (32.5)	18 (40.0)	7 (21.9)	0.138
Ustekinumab	20 (26.0)	12 (26.7)	8 (25.0)	1.000
Infliximab	18 (23.4)	15 (33.3)	3 (9.4)	0.016
Adalimumab	24 (31.2)	15 (33.3)	9 (28.1)	0.803
Vedolizumab	17 (22.1)	11 (24.4)	6 (18.8)	0.591
Hepatic steatosis	30 (39.0)	15 (33.3)	15 (46.9)	0.247
Cholecystolithiasis	10 (13.0)	4 (8.9)	6 (18.8)	0.304

Abbreviations: BMI, body mass index; EIMs, extraintestinal manifestations; OR, odds ratio; CI, confidence interval. Percentages may vary slightly across analyses due to missing data and case-wise exclusion.

**Table 5:** Multivariate binary regression for EIMs.

Variable	OR (95% CI)	*p* Value
Female sex	4.63 (1.04–20.63)	0.044
Perianal disease	0.04 (0.00–5.41)	0.201
Perianal fistula	0.37 (0.08–10.60)	0.366
Rectal stenosis	0.08 (0.01–0.90)	0.041
Autoimmune disease	23.50 (1.02–567.60)	0.049
Biologic therapy	1.63 (0.19–14.10)	0.657
Multiple biologics	20.11 (1.21–334.30)	0.036
Ustekinumab	4.05 (0.45–36.00)	0.210
Adalimumab	0.42 (0.05–3.89)	0.444

Only variables included in the final exploratory multivariable model are shown.

**Table 6:** Multivariate binary regression for Smoking.

Variable	OR (95% CI)	*p* Value
Female sex	0.35 (0.13–0.93)	0.036
Anastomosis stenosis	3.16 (1.17–8.53)	0.023
Inflammatory stenosis	0.22 (0.03–1.39)	0.106
Cholecystolithiasis	0.21 (0.04–1.18)	0.076

Only variables included in the final exploratory multivariable model are shown.

**Table 7:** Exploratory regression model for penetrating disease manifestations.

Variable	OR (95% CI)	*p* Value
Perianal disease	0.10 (0.01–4.13)	0.221
Small bowel fistula	11.82 (2.96–47.21)	<0.001
Perianal fistula	19.54 (4.63–82.51)	<0.001
Stenosis > 10 years	0.24 (0.07–0.78)	0.018
Abscess	1.65 (0.49–5.57)	0.422

Only variables included in the final exploratory multivariable model are shown.

**Table 8:** Multivariate binary regression for Hepatic Steatosis.

Variable	OR (95% CI)	*p* Value
Perianal disease	0.10 (0.01–4.13)	0.221
Intestinal fistula	28.91 (1.94–431.40)	0.015
Perianal fistula	10.01 (0.20–492.20)	0.247
Ileal stenosis	2.42 (0.43–13.51)	0.315
Intestinal resection	26.88 (1.02–709.00)	0.049
Appendectomy	17.43 (1.31–231.80)	0.030
Cholecystolithiasis	2.36 (0.20–28.39)	0.498

Only variables included in the final exploratory multivariable model are shown.

**Table 9:** Multivariate binary regression for rectal and ileal stenosis.

Variable	OR (95% CI) Rectal	*p* Value	Variable	OR (95% CI) Ileal	*p* Value
EIM	0.18 (0.02–1.67)	0.180	Female sex	2.84 (1.02–8.17)	0.050
Ileal stenosis	0.18 (0.04–0.82)	0.027	Rectal stenosis	0.17 (0.04–0.64)	0.009
Abscess	7.56 (0.73–78.80)	0.091	Biologic therapy	2.63 (0.95–7.28)	0.063
Perianal fistula	1.77 (0.33–9.55)	0.506	Hepatic steatosis	2.61 (0.87–7.77)	0.086

Only variables included in the final exploratory multivariable model are shown.

**Table 10:** Multivariate binary regression for abscess.

Variable	OR (95% CI)	*p* Value
BMI > 25 kg/m^2^	23.69 (1.23–454.90)	0.036
Infliximab	1.11 (0.12–10.57)	0.931
Perianal disease	30.18 (2.55–356.70)	0.007
Any fistula	27.30 (1.40–533.30)	0.029

Only variables included in the final exploratory multivariable model are shown.

## Data Availability

The data presented in this study are available on request from the corresponding author. The data are not publicly available due to privacy and ethical restrictions.

## Abbreviations

The following abbreviations are used in this manuscript:

BMI	Body mass index
CD	Crohn’s disease
CI	Confidence interval
CTE	Computed tomography enterography
EIMs	Extraintestinal manifestations
IBD	Inflammatory bowel disease
MRE	Magnetic resonance enterography
OR	Odds ratio
